# Eswatini Nursing Council Regulatory Reforms: Process towards Entry to Practice Examination

**DOI:** 10.5334/aogh.2800

**Published:** 2020-04-28

**Authors:** Glory Msibi, Nkosinathi Nkwanyana, Helen Kuebel

**Affiliations:** 1 Ministry of Health, SZ; 2 Eswatini Nursing Council, SZ; 3 Seed Global Health, Boston, Massachusetts, US

## Abstract

**Objective:**

To identify and to measure entry level competencies (knowledge, skills, attitudes, judgements) for nurses to practice safely and effectively in the Kingdom of Eswatini.

**Introduction:**

Eswatini, formerly known as Swaziland, is a small sub-Saharan country between South Africa and Mozambique. There are four nursing programs approved by the Eswatini Nursing Council (ENC) that provide nursing education in the areas of general nursing, midwifery, mental health and community health. The mandate of the ENC is to protect the public and to this end licensed nurses must be able to meet standardized entry level requirements.

**Methods:**

We identified gaps in expected competencies of new nurses led to comprehensive strategies by many stakeholders to close the gaps. Nursing competencies were categorized into seven learning domains with specific, measurable indicators included in each domain. Specific clinical skills essential for entry to practice were identified.

**Results:**

Provision of Quality Care; Information Management Systems; Emergency/Trauma/Disaster Management; Infection Prevention & Control; Leadership and Management; Ethics/Legal Issues/Professional Conduct; and Prevention/Treatment & Care of HIV, AIDS, TB are the seven competency domains that are measured on a newly developed standardized entry to practice multiple choice examination. Essential clinical skills are also assessed prior to obtaining licensure.

**Conclusion:**

Implementing these standards will ensure that nurses in Eswatini have the appropriate skill set to deliver care to their patients, improve their communities’ health, and enable the kingdom to make advances towards universal health coverage and attainment of the sustainable development goals.

## Introduction

The kingdom of Eswatini is a landlocked country in the East of Southern Africa bounded to the East by Mozambique and elsewhere by South Africa. (It should be noted that the name of the country was changed from Swaziland to Eswatini in 2018.) According to WHO Statistics 2016, the total population is 1,343,000, and population growth rate is 1.6% per annum [1]. The country is classified as a lower middle-income by the World Bank [2].

Life expectancy at birth is estimated at only 58 years (55 years for males, and 60 years for females) according to the WHO 2016 World Health Statistics. This shows significant improvement from the 2014 statistics and has been attributed to many factors, including the aggressive HIV and TB prevention and treatment programs as demonstrated by the political will of the government of Eswatini, the United States President’s Emergency Plan for AIDS Relief (PEPFAR) and other partners. As the country is facing current fiscal challenges, health remains a priority after education in the country with a total budget of 2.2 million Emalangeni in 2018. This shows the level of commitment the government of Eswatini has for the lives of its citizens.

There is a provision under leadership and governance through the Ministry of Health to strengthen regulatory bodies. One of these is the Eswatini Nursing Council (ENC). The ENC, like any other regulatory body in the world, has a mandate to protect the public by making sure the graduates that are produced by the training institutions have the required knowledge, skills, attitudes, and judgments to provide nursing and midwifery services to the populace. The entry-to- practice examinations support this mandate and will begin in August 2020 as a requirement for licensure.

## Methods

The process toward the entry-to-practice (ETP) examination has been a detailed and comprehensive journey, involving all stakeholders. In 2010 the ENC began the initial step by conducting a situational analysis to assess the knowledge and skills of the nurses and to identify potential gaps. The findings became the baseline as well as a point of departure for many projects. The projects included the introduction of the education and practice standards, entry-to-practice competences, ETP examination as well as reviewing the pre-service curricula for nursing and midwifery programs in Eswatini.

Strategies were developed to improve nursing standards and resulted in the publication in 2010 of *The Scope of Nursing Practice* [3]. Support was provided by PEPFAR/USAID through the Southern Africa Human Capacity Development (SAHCD) Coalition in Swaziland. This activity propelled the development of other regulatory frameworks, one of which was the entry-to-practice competencies. It should be noted that the 2010 *Scope of Nursing Practice* was reviewed and revised in 2019 and will be published by the ENC in 2020.

The competencies were developed with support from the African Health Regulatory Collaborative (ARC), PEPFAR/USAID, and the US Health Finance & Governance agency (HFG). Professor Cynthia Baker from the Canadian Nurses Association (CASNAS) was engaged as a consultant. A technical working group of 40 experts from both education and practice in Eswatini were assembled to work on the competencies. The goal of publishing competencies was to provide a detailed guide for education programs for nurses and midwives. The entry-to-practice nursing competencies were published by the Nursing Council in 2014 [4].

The ENC Competency Framework is client centred and includes four competency sets. The first, *General Nursing Competencies*, provide the outcome expectations for the three years of the basic nursing education that prepares students either to enter practice with a Registered Nurse diploma or to complete a baccalaureate degree in a specialty area in a fourth year of study. The three subsequent competency sets in the framework provide outcome expectations for specialization options that may be taken in the fourth year of a baccalaureate program or in a post diploma year of study. They are: midwifery, community health nursing, and community mental health nursing. Students enroll in one of these specializations after completing the three-year General Nursing program. Competencies may be defined as the combination of knowledge, skills, attitudes, and judgement the nursing graduate in Eswatini needs to practice effectively and safely on entering the workforce [5]. The Competency Framework has seven domains, and each competency is accompanied by a list of indicators. These list the the specific knowledge, attitudes, and skills students must learn if they are to integrate the competency into practice. The indicators provide measurable and observable manifestations of the competency.

The publication of the competencies followed a major curricula review completed by each of the nursing training institutions. The aim of the review was to identify gaps in terms of missing competencies and to revise curricula as needed. In 2016 the ENC approached ICAP at Columbia University (formerly known as the International Center for AIDS Care & Treatment Programs) for technical support for beginning the review of the curricula including HIV/AIDS. Judy Kanyola, a consultant from Kenya, was engaged for the exercise. The outcome of the review was competecy-based curricula for all the four practice areas: general nursing, midwifery, community health nursing, and community mental health. It is worth mentioning that all the four nursing training institutions were involved, and nurses from service delivery were part of the exercise. The curricula review marked the beginning of the final step of the reforms, which is introduction of the ETP examinations. To restate, the goal of the entry-to-practice examination is to improve the quality of health services provided by nurses and midwives who have been found competent and safe to practice before entering practice. This is also a means of gaining public trust on the nursing and midwifery profession as well as minimizing litigation by members of the public and lastly it will also help the ENC in screening international professional nurses who wish to work in the kingdom of Eswatini.

In 2017, the ENC solicited support from the United States Peace Corps/Seed Global Health to support the final phases of the project. Helen Kuebel, Nursing Education Consultant with Seed Global Health, a Boston-based NGO was engaged to provide technical support and coordinate the project. This process started with the development of a plan, expected activities, outcomes and timetable. The first step was to identify champions from each of the four nursing training institutions, who are: Dr. Colile Dlamini from the University of Eswatini (UNESWA), Hlengiwe Mohale from Southern Africa Nazarene University (SANU), Dr. Khombi Nkonde from Eswatini Medical Christian University (EMCU), and Sibonile Dlamini from Good Shepherd College of Nursing (GSCN). Additionally, ongoing support came from the Ministry of Health, Eswatini Nurses Association, and various nurse leaders. The core mandate of the champions was to coordinate faculty on each campus in moving this project ahead.

The next activity was for the champions to assist the ENC with the validation of the competencies in each curriculum of the various programs. The question was asked: In which course could the competency be identified as being included? This exercise was to ensure that when multiple choice questions were formulated based on the competencies, the competency can be identified in the curricula. The consultant worked on this with the institutions making sure that each of the indicators of the competencies is addressed in the curricula. This exercise meant numerous visits to the nursing institutions by the consultant and the ENC secretariat as well as planned meetings with the champions and faculty of the various programs. In some programs, curricula revisions were required because they had not included all the competencies needed in each of the seven domains.

The next step in the process was the formation of question (item writing) teams representing all nursing programs to write multiple choice questions for the ETP exam. The team leader and team members have clinical expertise in one of the four practice areas: general nursing, midwifery, mental health, and community health. The team leaders are Professor Tengetile R. Mathunjwa (general nursing), Dr. Oslinah B. Tagutanazvo (midwifery), Professor Nonhlanhla Sukati (mental health nursing), Faith Dlamini (community health nursing). Each of the team leaders is working diligently to foster a collegial community of mutual respect. They share creative ideas and strategies for nursing education while fulfilling their primary goals of writing multiple choice questions.

In August 2018, a comprehensive ETP workshop was held and covered the following topics:

1. Team-building exercises, including “The Marshmallow Challenge” [7]. Team building is important to enable nurses to learn how to work together toward mutual goals. The marshmallow challenge opened the door to “build a tower, build a team” and the importance of laughter and fun while establishing effective collaborative relationships.
2. Best practices for writing multiple choice questions [8].
3. Outlining principles of curriculum development including measurement of student outcomes and program outcomes [9,10,11].

## Results

The test blueprint for the ETP exams is based on seven comprehensive competency domains [6]. The competency domains are:

- The provision of quality care
- Information management systems
- Emergency, trauma, and disaster management
- Infection prevention and control
- Leadership and management
- Ethics, legal issues, and professional conduct
- Prevention, treatment, and care—HIV/AIDS and TB

In July 2019 an ETP pilot exam was conducted, and 99% of the 2019 graduates from all programs volunteered to participate. To help promote effective test review and feedback to each nursing program, Seed Global Health also funded a Scantron machine to generate detailed question analysis. The analysis looks at each question and provides information on how many test takers chose which distractor (wrong answer) in addition to how many chose the right answer. This assists in future evaluation of good/effective questions. The analysis further breaks down each question by domain so that each nursing program can identify strengths and weaknesses in their student outcomes and curriculum. The data is reported to each nursing program by numbers, not student names.

In addition to demonstrating nursing knowledge through the multiple-choice questions, the graduate or nurse seeking licensure in Eswatini must demonstrate selected essential clinical skills. To accomplish this goal, the ENC convened a group of nurse educators to develop the Standard Clinical Competencies Record Book (SCCRB) [12] that was published in 2018. Nurses and educators represented the four nursing training institutions, Mbabane Government Hospital, and Raleigh Fitkin Memorial Hospital. They listed essential skills for new nurses. Each student is required to perform essential procedures and record in the SCCRB. It is co- signed by clinical preceptors after the student has demonstrated competency in that procedure; the final mark is given by the lecturer. With the SCCRB, the graduate must demonstrate skills and abilities required for application of knowledge that the entry-to-practice exam measures. Similar to the pilot exams testing nursing knowledge, the implementation of the SCCRB was phased in starting in 2019, with required skills demonstrated by graduation in 2020 in order to obtain licensure.

## Conclusion

Implementing these standards will ensure that nurses in Eswatini have the appropriate skill set to deliver care to their patients, improve their communities’ health, and enable the kingdom to make advances towards universal health coverage and attainment of the sustainable development goals.
